# The Effect of Inclination on Spatiotemporal Gait Parameters in Special Forces Operators Under Tactical Load

**DOI:** 10.3390/jcm15093252

**Published:** 2026-04-24

**Authors:** Patryk Marszałek, Wojciech Paśko, Krzysztof Maćkała, Rafał Podgórski, Bartosz Dziadek, Natalia Jasińska, Élvio Rúbio Gouveia, Hugo Sarmento, Cintia França, Francisco Martins, Oliwia Król, Krzysztof Przednowek

**Affiliations:** 1 Faculty of Physical Culture Sciences, Collegium Medicum, University of Rzeszow, 35-959 Rzeszow, Poland; pmarszalek@ur.edu.pl (P.M.); wopasko@ur.edu.pl (W.P.); bdziadek@ur.edu.pl (B.D.); npoludniak@ur.edu.pl (N.J.); ok120237@stud.ur.edu.pl (O.K.); 2 Department of Track and Field, Wroclaw University of Health and Sport Sciences, 51-617 Wroclaw, Poland; krzysztof.mackala@awf.wroc.pl; 3 Department of Medicinal Chemistry and Metabolomics, Faculty of Medicine, University of Rzeszow, 35-959 Rzeszow, Poland; rpodgorski@ur.edu.pl; 4 Department of Physical Education and Sport, University of Madeira, 9020-105 Funchal, Portugal; erubiog@staff.uma.pt (É.R.G.); cintia.franca@staff.uma.pt (C.F.); joao.martins@staff.uma.pt (F.M.); 5 LARSyS, Interactive Technologies Institute, 9020-105 Funchal, Portugal; 6 Research Center in Sports Science, Health Sciences, and Human Development (CIDESD), 5000-801 Vila Real, Portugal; 7 CIPER, Faculty of Human Kinetics, University of Lisbon, 1649-004 Lisbon, Portugal; 8 Research Unit for Sport and Physical Activity (CIDAF), Faculty of Sports Sciences and Physical Education, University of Coimbra, 3004-504 Coimbra, Portugal; hugo.sarmento@uc.pt

**Keywords:** gait biomechanics, locomotor adaptation, load carriage, military personnel, overuse injury risk, special forces operators

## Abstract

**Background:** Special Forces Operators often carry out missions in conditions where the use of motor vehicles is impossible. Additional external load across areas with variable inclination may reduce walking efficiency and consequently limit the combat capability of soldiers. The aim of the study was to determine how ground inclination affects the spatiotemporal structure of gait in Special Forces Operators (SFO) with different military loads. **Methods:** The study included 50 operators from Polish special forces units. Measurements of walking were performed using the h/p/cosmos Gaitway 1D + 3D treadmill. Tests were conducted at four uphill inclination levels: 0%, 5%, 10%, and 15%. Each participant completed trials both without external load and with a 27 kg load (helmet, tactical vest, and backpack). Statistical analyses were performed using the Friedman test, the Durbin–Conover post hoc test, and linear mixed models (LMM) to assess interaction effects. The Robinson Symmetry Index (SI) was calculated to assess asymmetry between the dominant and non-dominant limbs. **Results:** Increasing inclination caused statistically significant changes in the spatiotemporal structure of gait. The greatest modifications were observed at 10–15% inclinations, particularly under the maximum load of 27 kg. A significant shortening of step length and gait cycle time was noted, while cadence showed a slight upward trend, especially at a 15% inclination with the highest load. Step width remained stable. **Conclusions:** Ground inclination, especially when combined with the additional mass of military equipment, significantly affects the locomotion of Special Forces Operators. The stable SI values and consistent step width indicate a high level of gait stability and effective adaptive mechanisms. However, the extent of spatiotemporal modifications observed at inclinations of 10–15% with a 27 kg load may increase the risk of overuse injuries among operators.

## 1. Introduction

Gait is the fundamental and natural form of human locomotion, providing autonomy and facilitating mobility throughout life [1,2]. It is a complex movement mechanism based on the integration of sensory and motor signals with the coordinated function of the entire musculoskeletal system [3,4]. During walking, the biomechanics of the human body resemble the operation of a well-balanced mechanism characterized by the absence of static stability while maintaining dynamic stability [5]. This requires coordinated activity of the lower limbs and a smooth transition from a stable bipedal stance to dynamic motion [6]. As a result, efficient gait determines overall physical performance and has a direct impact on movement efficiency, ergonomics, and comfort in all aspects of human functioning [7].

The efficiency of a soldier’s gait is influenced by several external factors, including footwear, clothing, and the additional load carried during military operations [8]. Among these factors, the total mass of military equipment remains the primary limitation to marching performance [9]. A military load of approximately 20 kg leads to increased oxygen consumption, indicating higher energy demands and greater physiological strain [10]. Prolonged load carriage also results in muscle fatigue, particularly affecting the knee flexors and extensors [11]. Furthermore, it has been demonstrated that energy expenditure in Special Forces Operators during movement with military load may increase by as much as 20–27% [12]. Consequently, carrying such a load also induces changes in the kinetics and kinematics of gait in SFO, leading to increased ground reaction forces (GRF), alterations in joint range of motion, and the occurrence of minor asymmetries [13,14,15,16]. These adaptations are also reflected in spatiotemporal parameters, including reduced preferred step length, decreased ground contact time, and increased cadence [11,14,17,18,19].

Maintaining efficient and stable gait under such heavy load conditions is therefore a key determinant of soldiers’ operational effectiveness [18,20]. To reduce the negative biomechanical effects of load carriage, specialized systems such as shoulder straps and hip belts have been developed to improve comfort and walking efficiency during long-distance marches by redistributing mechanical loads and reducing local strain and fatigue [21].

An important determinant of gait efficiency under combat conditions is terrain inclination, which significantly modifies a soldier’s locomotion pattern, particularly when combined with heavy load carriage [22]. Military movement typically occurs across varied terrain, where the use of mechanized transport is often limited or entirely impractical [23]. Although human gait patterns are naturally adaptable to different environmental conditions, ground inclination introduces an additional challenge for the neuromuscular system [24]. Moreover, terrain slope combined with external load has been identified as one of the main factors increasing the risk of overuse injuries among soldiers [25]. According to Molloy et al. [26], 27% of overuse injuries were attributed to prolonged and fatiguing marches. Previous studies also indicate that joint compression forces tend to increase with varying terrain gradients, thereby imposing greater mechanical stress on the musculoskeletal system [27].

Despite the growing interest in military load carriage and its physiological implications, the biomechanics of soldiers’ gait remain relatively underexplored [28]. In a recent systematic review, Walsh et al. [28] confirmed that out of 20 included publications, only two investigated soldier gait across different terrain inclinations [28,29,30]. Notably, none of the included studies focused on elite military personnel, such as Special Forces Operators (SFO). Understanding how terrain inclination interacts with external load to influence gait biomechanics is crucial for preventing musculoskeletal injuries and improving marching efficiency among soldiers [28].

Due to the specificity of their duties, SFO are characterized by above-average physical and psychomotor preparedness, which may classify them as a distinct military subgroup in terms of movement control and locomotor adaptations [31,32,33,34]. It has been hypothesized that an increase in uphill inclination, combined with an external load, will significantly alter gait parameters, and that SFO may employ adaptive strategies to mitigate the severity of biomechanical disturbances during locomotion. Therefore, the present study aimed to determine how terrain gradient, in combination with military load carriage, affects the gait pattern of SFO.

## 2. Material and Methods

### 2.1. Material

The study was conducted on a group of 50 Special Forces Operators, who had completed nearly a year-long training program consisting of both selection and basic preparation phases [35]. Soldiers were selected as an elite population for whom marching with external load in challenging terrain conditions constitutes an integral element of military service. The group was carefully selected in terms of body height and mass to minimize confounding variables and ensure the reliability of the obtained results. All participants provided written informed consent prior to testing and underwent a comprehensive medical assessment. Individuals with recent musculoskeletal injuries within the past 6 months (particularly affecting the lower limbs), neurological disorders, balance impairments, or any conditions that could affect gait, stability, or load carriage ability were excluded from participation. The characteristics of the study group are presented in Table 1.

The conducted research was part of a project implemented by the University of Rzeszow entitled “Identification and Monitoring of Health Parameters in Soldiers and Uniformed Service Officers”, which received a positive opinion from the Bioethics Committee of the University of Rzeszow (approval no. 2023/12/0060). All procedures involving human participants were carried out in accordance with the ethical standards of the Ethics Committee of the University of Rzeszow and with the 1964 Declaration of Helsinki and its later amendments.

### 2.2. Methods

The study was conducted in the Motion Analysis Laboratory at the University Athletic Center–Innovative Research in Sport Facility, University of Rzeszow. The first stage of the study involved measuring body height using a Seca 213 stadiometer (Seca GmbH & Co. KG, Hamburg, Germany). After assuming the Frankfurt anatomical position, each participant was instructed to take a maximal inhalation, after which the maximum value indicated on the device scale was recorded. The next stage consisted of body composition analysis, performed using a bioelectrical impedance analyzer (InBody 770, InBody Co., Ltd., Seoul, Republic of Korea).

The main stage of the study involved gait analysis, which was performed using an advanced Gaitway 3D and 1D Pressure/Force Treadmill (h/p/cosmos, Nussdorf-Traunstein, Germany) with a belt surface of 190 × 65 cm, operating within a speed range of 0–40 km/h and an inclination range from −20% to +20%. The treadmill allows for precise measurement of individual gait phases and spatiotemporal parameters under varying speeds and inclinations. Data analysis was conducted using Noraxon MR3 software (version 3.18.10, Noraxon USA, Inc., Scottsdale, AZ, USA). The technologically integrated treadmill enabled the acquisition of accurate biomechanical parameters for both lower limbs. The following spatiotemporal gait parameters were analyzed: stride length, step length, stride time, step time, cadence, step width, double support, stance phase, single support, load response, pre-swing, and foot rotation.

The experimental procedure involved walking at a constant speed of 5.5 km/h. This velocity was selected as it lies between the average adult walking speed of 5 km/h [36] and the standard military marching speed of 6 km/h [37]. Each participant completed two trials for each of four uphill inclinations: 0%, 5%, 10%, and 15%.

**Figure 1:**
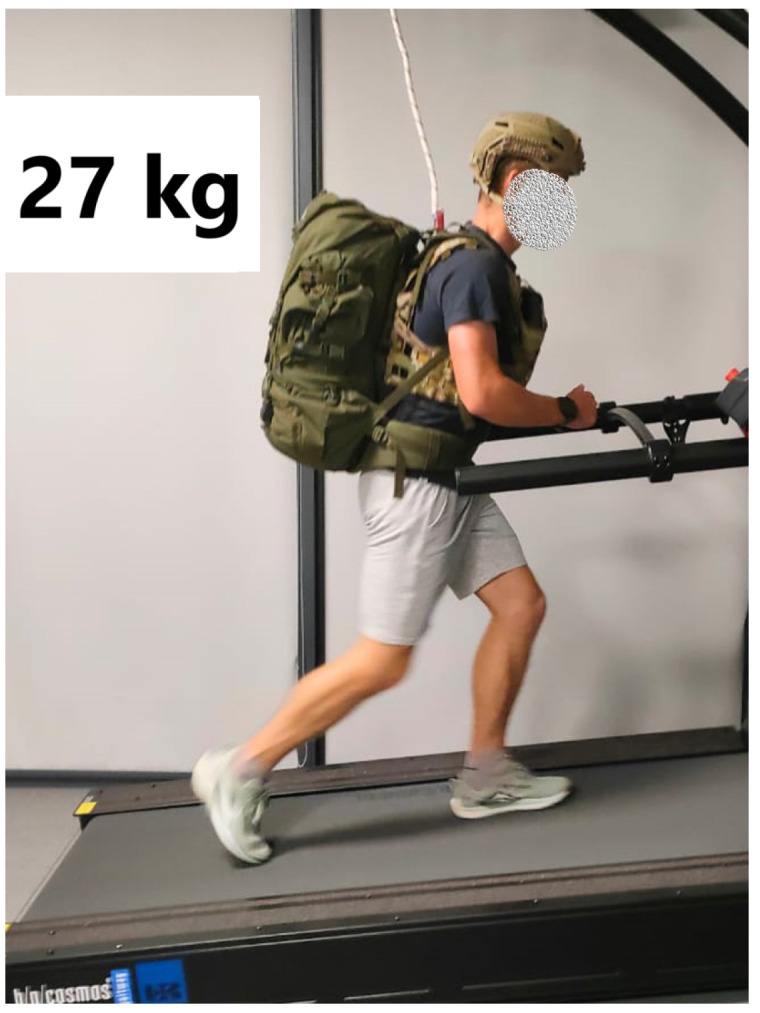
Gait test under load conditions. Source: author’s own elaboration.

The gait measurements were performed while participants wore sports attire and completed trials under two load conditions: without external load and with an external load of 27 kg, consisting of a tactical vest (6 kg), a helmet (1 kg), and a backpack (20 kg). The configuration of the load during the marching trials is illustrated in Figure 1. Data recording lasted 20 s and commenced after the treadmill reached the target speed and participants completed an additional 10 s adaptation period, ensuring stabilization of the gait pattern. In cases of stumbling or difficulty in achieving initial adaptation, the trial was repeated.

### 2.3. Statistical Analysis

Statistical analyses were performed using Statistica 13.1 and R version 4.3.3. Descriptive statistics including sample size, arithmetic mean, median, minimum and maximum values, standard deviation, and coefficient of variation were used to characterize the study group and selected spatiotemporal parameters.

The normality of data distribution was verified using the Shapiro–Wilk test. Consequently, the Friedman test was applied as a nonparametric alternative to repeated measures ANOVA, followed by the Durbin–Conover post hoc test for unilateral parameters. The Friedman test was used to identify statistically significant differences between various conditions of terrain inclination and external load, while the Durbin–Conover post hoc test allowed for the determination of specific inclination and load levels that exhibited statistically significant differences. Additionally, linear mixed models (LMM) were used to examine the interaction between inclination and external load for the analyzed gait parameters.

The results of spatiotemporal parameters were divided into bilateral and unilateral categories. To identify lower-limb asymmetry in bilateral parameters, the Robinson Symmetry Index (SI) was calculated, as it is one of the most commonly used indicators for assessing asymmetry in gait kinematic parameters [38]. The lower limbs were categorized as dominant and non-dominant based on the lateralization of each participant. The SI values were computed using the following equation:

$$SI=\frac{2\cdot (X_{\mathrm{dominant}\mathrm{limb}}-X_{\text{non-dominant}\mathrm{limb}})}{X_{\mathrm{dominant}\mathrm{limb}}+X_{\text{non-dominant}\mathrm{limb}}}\cdot 100\%$$

The magnitude of the *SI* enabled the identification of kinematic differences in lower limb performance for individual parameters. Positive or negative *SI* values indicated the direction of asymmetry.

## 3. Results

Table 1 presents the numerical characteristics of the examined group. The mean age of the operators was 35.91 years. The mean body height was 179.85 cm, with extreme values ranging from 169 cm to 191 cm. The median height was similar to the mean, and the coefficient of variation was 3.72%. The mean body mass reached 86.00 kg, while the mean Body Mass Index (BMI) was 26.59 $\frac{\mathrm{kg}}{m^{2}}$. The mean values of Fat-Free Mass (FFM) and Skeletal Muscle Mass (SMM) were 72.19 kg and 41.40 kg, respectively, whereas body fat content ranged from 10.40% to 24.60%.

In the analysis of unilateral parameters, the following variables were included: double support, step width, stride length, stride time, and cadence. Under the zero-load condition (Table 2), the Friedman test revealed statistically significant differences (*p* < 0.05) between the different uphill inclination conditions (0%, 5%, 10%, and 15%) for all analyzed parameters, except for step width, with a gradual increase in double support and slight changes in stride length, stride time, and cadence, while step width remained stable. A moderate effect size was observed only for the double support parameter (e.s = 0.30). The post hoc Durbin–Conover analysis indicated statistically significant differences (*p* < 0.01) between all tested conditions, except for the comparison between 10% and 15% inclinations, where no statistical significance was found. For the remaining parameters, the effect size did not exceed 0.10, suggesting a minimal influence of terrain inclination during trials performed in sportswear without additional military load. Comparison of individual test conditions using the Durbin–Conover test revealed significant differences only between 0% and 5% inclinations and between 10% and 15% inclinations.

The effect of treadmill inclination during trials performed with a tactical load of 27 kg resulted in statistically significant differences (*p* < 0.001) across all analyzed unilateral parameters, except for step width, with an increase in double support, a reduction in stride length and stride time, and an increase in cadence, while step width remained stable. The Kendall’s W coefficient of concordance for the examined parameters ranged from 0.14 (stride length) to 0.30 (stride time and cadence), indicating a low to moderate effect size of the observed changes. For stride time, cadence, and stride length, the post hoc analysis revealed statistically significant differences (*p* < 0.001) only in comparisons involving the 15% inclination condition. In contrast, for the double support parameter, significant differences were observed for all changes in treadmill inclination, except for the transition from 5% to 10%.

The analysis of bilateral parameters (Table 3) under the 0 kg load condition revealed that treadmill inclination generated statistically significant differences (*p* < 0.001) across various phases and subphases of the gait cycle. The results indicated that increasing inclination led to a prolongation of the stance phase. A similar trend was observed for the load response and pre-swing phases, whose relative durations increased with higher inclinations. Only the single support phase showed a decrease from 37.30% to 36.61% for the dominant limb and from 37.15% to 36.54% for the non-dominant limb. Both step length and step time exhibited only minor changes, reflected in a small effect size not exceeding 0.1. Although statistically significant differences were observed, their magnitude remained low. The only parameter that did not show statistically significant differences across the analyzed inclination conditions was foot rotation.

Changes in the spatiotemporal structure of gait resulting from increased treadmill inclination included prolongation of the stance phase, increases in load response and pre-swing, and a decrease in the single support phase. A distinct prolongation of the stance phase was noted, with the highest values reaching 65.38 ± 0.38% for the dominant limb and 65.10 ± 1.25% for the non-dominant limb. Among the analyzed subphases, load response and pre-swing phases increased in duration, while the single support phase decreased. Step length was reduced by approximately 2 cm, and step time decreased from 512.02 to 497.93 ms for the dominant limb and from 512.84 to 500.21 ms for the non-dominant limb. The maximum values of Kendall’s W coefficient were 0.36 for the dominant limb and 0.28 for the non-dominant limb, indicating moderate differences.

In the group of unilateral parameters, the LMM analysis (Table 4) revealed a statistically significant interaction between external load and inclination for stride length, stride time, and cadence (*p* < 0.001). With increasing load and inclination, a reduction in both stride length and stride time was observed, accompanied by an increase in cadence. In contrast, no significant interaction effects were found for step width and double support, and these parameters remained relatively stable across all conditions. For bilateral parameters, significant interaction effects were identified for step length and step time in both the dominant and non-dominant limbs. With increasing load and inclination, both parameters showed a progressive decrease. No significant interaction effects were observed for the remaining parameters, including stance phase, load response, single support, pre-swing, and foot rotation. These parameters exhibited only minor, systematic changes under the combined influence of both factors, and their overall contribution was limited.

**Figure 2:**
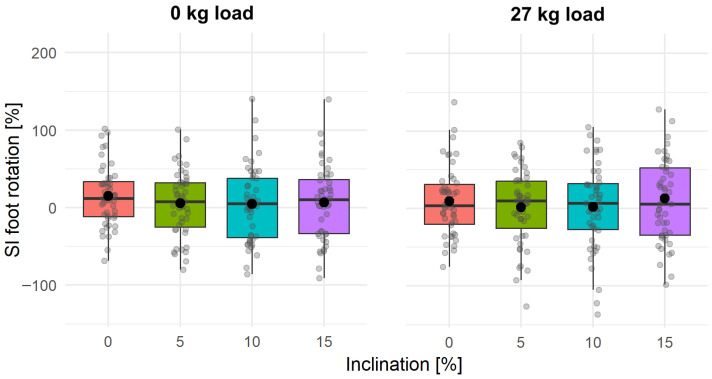
The value of Robinson’s Symmetry Index (SI) for foot rotation under the analyzed inclination conditions. (**Left**) panel: no external load (0 kg); (**right**) panel: external load (27 kg).

Figure 2 presents the SI values for foot rotation across the analyzed test conditions. Among all examined parameters, the SI for foot rotation exhibited the greatest variability. The observed data dispersion reflected individual biomechanical strategies regarding both the direction and magnitude of rotation, which is typical for angular parameters. The mean SI values ranged from 14.19% to 29.92% and remained relatively stable across all analyzed conditions.

The post hoc analysis revealed statistically significant differences only in the trials without additional external load (*p* < 0.05). Moreover, the results indicated that a significant reduction in foot rotation occurred during unloaded trials performed at 10% and 15% treadmill inclinations. However, the Kendall’s W coefficient indicated a low effect size (*W* = 0.07). For the remaining parameters, no statistically significant differences were observed, and the maximum absolute SI value was approximately 1%.

## 4. Discussion

In the present study, it was observed that the interaction between treadmill inclination and external load influenced selected spatiotemporal gait parameters. The LMM analysis confirmed that interaction effects were primarily present in parameters related to the regulation of stride length and gait rhythm, whereas no significant interaction effects were found for the remaining variables. However, the direction and magnitude of these changes depended not only on the degree of inclination but also on the mass of the carried tactical equipment. During walking without load, a slight increase in step length was recorded along with increasing surface inclination, which is consistent with the observations of other authors among healthy individuals not associated with military service [39,40,41,42]. An opposite trend was observed among SFO during walking with a 27 kg load at a 15% inclination, where step length was reduced by nearly 4 cm. Similar results at a 10.5% inclination among soldiers were reported by Fellin et al. [29]. Under loading conditions of 20–40 kg, however, the researchers recorded only a slight reduction in step length, not exceeding 3 cm. Interestingly, even an increase in external load did not generate significant differences in the spatiotemporal structure of gait. It should be emphasized, however, that the studied group had completed basic training and had prior experience in carrying additional military equipment. Much greater differences were reported by Kimel et al. [43], who found a reduction of a single step by 12% and of the entire gait cycle by as much as 13%. In their study, however, a higher inclination (17.6%) was used and no additional load was applied. The results of the present study, along with findings from other authors [43], indicate that only an inclination of approximately 15% or greater may significantly increase changes in the spatiotemporal structure of gait.

According to previous studies, cadence is the main adaptive parameter in response to changes in terrain inclination [28]. In our study, a 15% inclination caused a significant increase in step frequency, particularly under a 27 kg load. A similar effect among soldiers was observed by Fellin et al. [29]. In contrast, Strutzenberger et al. [40] reported no increase in cadence, which may have resulted from a decrease in walking speed. The interaction between shortened step length and increased cadence at a constant walking velocity may reflect an adaptive biomechanical strategy that helps reduce the impact of gravitational forces and lower the load on the musculoskeletal system [22,44,45].

The observed changes in the spatiotemporal structure of gait among the operators also involved the proportions of individual gait cycle phases, with particular attention to the subphases of the stance phase. However, these differences did not exceed 1%, which is consistent with the findings of Vieira et al. [46], who also reported minor shifts in gait phase proportions at a 10% inclination. Similar results were presented by Tulchin et al. [47], indicating that the prolongation of the stance phase was accompanied by an increase in ankle joint flexion angle.

In the subphase analysis, it was observed that the loading response and push-off durations slightly increased with greater inclination, whereas the single support phase was shortened. Although these changes were minor, they may indicate an increased need for stabilization and generation of propulsive force under more demanding walking conditions. These findings are consistent with those reported by Lay et al. [44] and Alexander et al. [48], who described kinematic modifications in lower-limb joints during uphill walking, suggesting greater activation of postural muscles during the stance phase. Another important parameter was the double support time, which increased by up to 1.5% between the 0% and 15% inclinations. These results align with the observations of Strutzenberger et al. [39] and Kimel et al. [43], who also noted a similar increase in the proportion of this phase. Fellin et al. [29] emphasized that an increased double support phase represents a compensatory strategy adopted by soldiers marching with a 20 kg load. It can therefore be assumed that the inclusion of additional support time plays a crucial role in maintaining locomotor efficiency and stability under the increasing biomechanical demands associated with terrain inclination.

A key parameter in assessing dynamic gait stability and biomechanical adaptation to changing external conditions is step width [49,50,51]. In the present study, no significant changes were observed in the mean step width, which may be attributed to the high level of training and motor control among SFO. Similar results among healthy individuals were reported by Castano et al. [41], who also found no increase in step width. However, the authors noted a marked increase in the variability of this parameter across different inclinations, suggesting that the mean step width value does not always reflect the actual adaptive mechanisms involved [41,52,53].

The findings of the present study indicate that among SFO, the standard deviation of step width remained stable regardless of treadmill inclination. This stability may reflect high adaptive capacity and an above-average level of motor preparedness in this group compared to the civilian population.

The literature also emphasizes the importance of symmetry indices in assessing gait stability [54]. Among SFO, the Robinson SI did not exceed 1%, which is below the lowest normative values reported by Blazkiewicz et al. [55]. Moreover, within the studied group, the SI value remained stable regardless of the testing conditions. The low SI values and minimal variability in step width may therefore reflect the high adaptive capacity and superior motor preparedness of the SFO group. This interpretation is supported by previous findings from studies conducted among athletes, indicating that a higher level of motor preparedness is associated with lower kinematic asymmetry, more optimized movement patterns, and lower kinematic variability [56,57]. Similar adaptive mechanisms may also occur in Special Forces Operators.

In the present study, significant changes were observed in the spatiotemporal parameters that are considered key predictors of overuse injuries in the literature. Particular attention should be given to the stance phase, as its prolongation is associated with greater shock absorption and step stabilization. Springer et al. [37] indicated that a loading phase duration exceeding 12.15% of the gait cycle constitutes a strong predictor of injuries within the knee and ankle joints. In the current study, this phase was prolonged, which may suggest the presence of effective adaptive mechanisms aimed at reducing musculoskeletal load. Another important aspect is step variability, which reflects dynamic stability and the ability to adapt to changing conditions. According to the literature, greater step time variability is considered a significant risk factor for lower-limb injuries [37,58]. In the present study, standard deviations remained stable regardless of treadmill inclination or external load, which may indicate a high level of motor preparedness among SFO.

In this study, one of the parameters that did not show statistically significant differences between the analyzed slope conditions, regardless of the applied load, was foot rotation. At the same time, relatively high variability in the symmetry index (SI) was observed. This result is consistent with the findings of Rožac et al. [59], who demonstrated that increasing load in professional police officers does not lead to an increase in spatiotemporal asymmetry, and foot rotation remains one of the most stable spatial parameters. In contrast, Dewolf et al. [60] indicated that gait adaptation to slope occurs primarily through changes in global orientation and functional limb length, while maintaining overall intersegmental coordination. In light of these observations, it can be assumed that biomechanical adaptations for this parameter are global in nature and involve the entire lower limb, while maintaining intersegmental coordination. In this case, the foot does not constitute the primary adaptive mechanism, but rather reflects an individual locomotor strategy, which may explain its stability at the group mean level and relatively high variability at the inter-individual asymmetry level.

The high level of adaptive capacity observed among SFO may be partially related to the age and military experience of the studied group. Molloy et al. [26] emphasized that during the initial phase of military service, the risk of overuse injuries is significantly higher. In the study by Springer et al. [37], 31.6% of recruits sustained overuse injuries during their first year of training, despite being young and physically fit men. Moreover, this group exhibited a pronounced step length asymmetry (3.45 ± 2.72). In contrast, the mean age of SFO in the present study was nearly 35 years, while in the study by Paśko et al. [61], candidates for SFO averaged 30 years of age. These data suggest that professional experience among operators may be associated with greater gait stability and a lower risk of injury, particularly under conditions of high inclination and additional load.

The conducted study showed that the inclination of the surface significantly affects the spatiotemporal structure of soldiers’ gait. Even greater importance for the biomechanics of operators’ locomotion was observed for the additional military load, consisting of a helmet, tactical vest, and a backpack with a total mass of 27 kg. Particularly pronounced modifications of the parameters were observed when both factors were combined, especially at inclinations of 10–15%. Special Forces Operators, however, demonstrated high step stability and maintained symmetry, while the observed kinematic changes were gradual and adaptive to the increasing biomechanical demands. It should be noted that even small kinematic changes may have practical relevance, particularly in terms of increased physiological load and energy expenditure under demanding operational conditions.

### Study Limitations

One of the main limitations of this study is the use of treadmill walking, which differs from movement on natural terrain, as the stationary surface and belt-driven motion may partially reduce the stability demands encountered in real tactical terrain. Another limitation is the use of athletic shoes, which differ significantly from military footwear and may affect the gait kinematics of soldiers [62]. Future studies should therefore include military footwear and clothing, which may modify the soldiers’ movement patterns. Another aspect that has not been examined is locomotion with individual long firearms. Although carrying systems allowing for hands-free transport of assault rifles are commonly used, they are designed to be operated with both upper limbs [63]. Consequently, some operational tasks requiring the rifle to be held with both hands may disturb the gait biomechanics of soldiers.

Organized military marching, however, does not allow movement at an individual pace, as soldiers must operate collectively and adjust their speed to other team members. It should be noted that walking speed may vary; therefore, future studies should include different velocities and diverse terrain profiles. Moreover, such analyses should be conducted under field conditions, which may be crucial for understanding biomechanical changes in the spatiotemporal structure of gait among SFO.

## 5. Conclusions

The conducted study demonstrated that the inclination of the surface significantly modifies the spatiotemporal structure of gait among Special Forces Operators. The greatest changes were observed when both factors were combined, particularly at inclinations of 10–15% and with an external load of 27 kg, which was manifested by a shortening of step length, changes in gait cycle time, and a prolongation of the stance phase. Although this study did not allow for the determination of a clear injury threshold, a 15% incline combined with a 27 kg load resulted in the most pronounced and consistent biomechanical adaptations, suggesting that this condition may serve as a practical reference point for cautious progression in weighted march training. Special Forces Operators, however, showed high step stability and a low level of asymmetry, indicating their above-average adaptive capabilities. The observed kinematic modifications may represent a compensatory strategy that increases locomotor stability but also indicates a higher risk of overuse injuries. The results suggest that training programs should include marching with progressive loading in field conditions with variable terrain inclinations. Such an approach may reduce the risk of overuse injuries and improve the effectiveness of biomechanical adaptation among soldiers. The analysis of the body composition of Special Forces Operators suggests that a high level of motor preparedness supports the development of adaptive mechanisms that enable efficient marching. Therefore, exercises aimed at increasing strength should be a permanent element of military training. These results indicate that training programs should incorporate a gradual increase in load and terrain incline, with particular caution in the 10–15% range, where the physical demands become more pronounced.

## Figures and Tables

**Table 1:** Characteristics of the study group.

	$\bar{x}$	Me	Min	Max	sd	V
Age (years)	35.91	35.00	26.00	51.00	6.56	18.27
Body height (cm)	179.85	178.60	169.00	191.00	6.69	3.72
Body mass (kg)	86.00	86.10	72.20	101.80	7.58	8.81
BMI ($\frac{\mathrm{kg}}{m^{2}}$)	26.59	26.50	23.10	30.60	1.96	7.38
TBW (kg)	52.80	52.10	43.60	64.10	5.19	9.83
Fat mass (kg)	13.83	13.30	7.90	19.50	3.28	23.71
FFM (kg)	72.19	71.20	59.70	87.40	7.09	9.82
SMM (kg)	41.40	41.60	33.80	50.10	4.32	10.43
FAT (%)	16.17	15.70	10.40	24.60	3.71	22.94

BMI—Body Mass Index, TBW—Total Body Water, Fat mass—Fat tissue mass, FFM—Fat Free Mass, SMM—Skeletal Muscle Mass, FAT —Body Fat Percentage, $\bar{x}$—arithmetic mean, Me—median, min—minimum value, max—maximum value, sd—standard deviation, V—coefficient of variation [%].

**Table 2:** Analysis of unilateral gait parameters under different inclination conditions with a load of 0 kg and 27 kg.

						Post-Hoc Analysis			
**Parameter**	**IN**	$\bar{x}$	**sd**	p	**e.s**	**0%**	**5%**	**10%**	**15%**
**Double support [%]**									
Load of 0 kg	0%	25.56	1.77	<0.001	0.30	x	***	***	***
	5%	26.23	1.57			***	x	**	***
	10%	26.62	1.83			***	**	x	NS
	15%	26.85	1.78			***	***	NS	x
Load of 27 kg	0%	29.36	1.72	<0.001	0.22	x	***	***	***
	5%	29.97	1.98			***	x	NS	*
	10%	30.14	1.83			***	NS	x	**
	15%	30.48	2.35			***	*	**	x
**Step width [cm]**									
Load of 0 kg	0%	11.34	2.92	0.14	0.04	x	NS	NS	NS
	5%	11.77	2.75			NS	x	NS	NS
	10%	12.10	2.87			NS	NS	x	NS
	15%	12.20	3.05			NS	NS	NS	x
Load of 27 kg	0%	12.48	2.77	0.48	0.02	x	NS	NS	NS
	5%	12.51	2.84			NS	x	NS	NS
	10%	12.95	2.90			NS	NS	x	NS
	15%	12.95	2.73			NS	NS	NS	x
**Stride length [cm]**									
Load of 0 kg	0%	157.67	6.34	<0.05	0.06	x	NS	**	NS
	5%	159.11	6.99			NS	x	NS	NS
	10%	159.64	7.75			**	NS	x	*
	15%	158.42	8.09			NS	NS	*	x
Load of 27 kg	0%	155.52	6.38	<0.001	0.14	x	NS	NS	***
	5%	156.20	7.05			NS	x	NS	***
	10%	155.35	7.76			NS	NS	x	***
	15%	151.39	8.13			***	***	***	x
**Stride time [ms]**									
Load of 0 kg	0%	1038.74	41.50	<0.05	0.07	x	NS	**	NS
	5%	1046.79	45.98			NS	x	NS	NS
	10%	1051.09	50.74			**	NS	x	*
	15%	1043.01	52.55			NS	NS	*	x
Load of 27 kg	0%	1024.86	42.21	<0.001	0.30	x	NS	NS	***
	5%	1028.84	46.96			NS	x	NS	***
	10%	1023.33	51.60			NS	NS	x	***
	15%	998.14	54.01			***	***	***	x
**Cadence [steps/min]**									
Load of 0 kg	0%	115.72	4.56	<0.05	0.07	x	NS	**	NS
	5%	114.87	4.88			NS	x	NS	NS
	10%	114.45	5.46			**	NS	x	*
	15%	115.37	5.75			NS	NS	*	x
Load of 27 kg	0%	117.30	4.76	<0.001	0.30	x	NS	NS	***
	5%	116.90	5.25			NS	x	NS	***
	10%	117.59	5.87			NS	NS	x	***
	15%	120.61	6.63			***	***	***	x

IN—inclination; $\bar{x}$—mean; sd—standard deviation; *p*—Friedman test; e.s—effect size (Kendall’s *W*); x—same-condition comparison; NS—not significant; *—p<0.05; **—p<0.01; ***—p<0.001.

**Table 3:** Characteristics of selected gait phases at various inclinations with a load of 0 kg and 27 kg.

		Dominant Limb				Non-Dominant Limb			
Parameter	IN	$\bar{x}$	sd	p	e.s	$\bar{x}$	sd	p	e.s
**Stance phase [%]**									
Load of 0 kg	0%	62.84	0.91	<0.001	0.31	62.70	0.97	<0.001	0.26
	5%	63.20	0.85			63.02	0.85		
	10%	63.35	1.01			63.25	0.92		
	15%	63.46	1.04			63.39	0.84		
Load of 27 kg	0%	64.75	0.98	<0.001	0.26	64.59	0.85	<0.001	0.20
	5%	65.04	1.06			64.89	1.02		
	10%	65.18	1.03			64.95	0.94		
	15%	65.38	1.20			65.10	1.25		
**Load response [%]**									
Load of 0 kg	0%	12.79	0.93	<0.001	0.29	12.77	0.96	<0.05	0.27
	5%	13.12	0.87			13.11	0.82		
	10%	13.34	0.95			13.28	1.01		
	15%	13.40	0.98			13.46	0.95		
Load of 27 kg	0%	14.64	0.91	<0.001	0.28	14.73	0.92	<0.001	0.15
	5%	14.95	1.10			15.01	1.01		
	10%	15.07	1.01			15.07	0.98		
	15%	15.21	1.26			15.26	1.23		
**Single support [%]**									
Load of 0 kg	0%	37.30	0.96	<0.001	0.25	37.15	0.91	<0.001	0.31
	5%	36.98	0.84			36.81	0.85		
	10%	36.75	0.91			36.64	1.02		
	15%	36.61	0.84			36.54	1.05		
Load of 27 kg	0%	35.41	0.85	<0.001	0.22	35.23	0.98	<0.001	0.21
	5%	35.11	1.03			34.94	1.08		
	10%	35.05	0.93			34.85	1.03		
	15%	34.91	1.24			34.64	1.21		
**Pre swing [%]**									
Load of 0 kg	0%	12.78	0.96	<0.001	0.25	12.79	0.92	<0.001	0.29
	5%	13.11	0.82			13.12	0.88		
	10%	13.28	1.02			13.35	0.96		
	15%	13.46	0.96			13.41	1.00		
Load of 27 kg	0%	14.72	0.92	<0.001	0.19	14.64	0.91	<0.001	0.27
	5%	15.03	1.01			14.96	1.09		
	10%	15.08	0.97			15.08	1.01		
	15%	15.26	1.23			15.22	1.25		
**Step length [cm]**									
Load of 0 kg	0%	78.75	3.61	<0.05	0.05	78.91	3.05	<0.01	0.08
	5%	79.50	3.80			79.59	3.53		
	10%	79.73	4.21			79.90	3.83		
	15%	79.19	4.11			79.24	4.21		
Load of 27 kg	0%	77.84	3.48	<0.001	0.34	77.68	3.14	<0.001	0.28
	5%	78.24	3.75			77.94	3.53		
	10%	77.62	3.88			77.72	4.06		
	15%	75.76	4.11			75.64	4.21		
**Step time [ms]**									
Load of 0 kg	0%	518.57	21.47	0.07	0.05	520.16	21.02	<0.05	0.08
	5%	522.44	23.98			524.35	23.05		
	10%	524.73	26.46			526.36	25.27		
	15%	521.44	28.09			521.55	25.32		
Load of 27 kg	0%	512.02	22.17	<0.001	0.36	512.84	21.07	<0.001	0.26
	5%	513.99	25.25			514.85	22.97		
	10%	510.58	27.06			512.75	25.70		
	15%	497.93	27.97			500.21	26.94		
**Foot rotation [°]**									
Load of 0 kg	0%	8.78	2.90	0.43	0.01	7.46	3.68	0.36	0.02
	5%	8.54	3.02			7.66	3.70		
	10%	8.08	3.19			7.45	3.66		
	15%	8.51	3.52			7.55	3.77		
Load of 27 kg	0%	7.56	2.77	0.58	0.01	6.63	3.58	0.61	0.01
	5%	7.19	2.97			6.57	3.69		
	10%	7.20	3.27			6.49	3.68		
	15%	7.53	2.91			6.76	3.60		

IN—inclination; $\bar{x}$—mean; sd—standard deviation; *p*—Friedman test; e.s—effect size (Kendall’s *W*).

**Table 4:** LMM results for the interaction effect (IN × OZ) on spatiotemporal gait parameters.

Unilateral Parameters						
**Parameter**	**F**		p	$\eta_{p}^{2}$		
Double support [%]	0.49		0.69	0.004		
Step width [cm]	0.43		0.73	0.004		
Stride length [cm]	13.59		<0.001	0.106		
Stride time [ms]	12.68		<0.001	0.100		
Cadence [steps/min]	13.07		<0.001	0.103		
**Bilateral Parameters**						
	**Dominant Limb**			**Non-Dominant Limb**		
**Parameter**	**F**	p	$\eta_{p}^{2}$	**F**	p	$\eta_{p}^{2}$
Stance phase [%]	0.20	0.90	0.002	0.83	0.48	0.007
Load response [%]	0.27	0.85	0.002	0.63	0.60	0.005
Single support [%]	0.95	0.42	0.008	0.37	0.78	0.003
Pre swing [%]	0.57	0.63	0.005	0.31	0.82	0.003
Step length [cm]	11.43	<0.001	0.091	11.30	<0.001	0.090
Step time [ms]	14.67	<0.001	0.114	8.75	<0.001	0.071
Foot rotation [°]	0.98	0.40	0.009	0.41	0.75	0.004

F—test statistic; *p*—significance level; $\eta_{p}^{2}$—partial eta squared.

## Data Availability

The data presented in this study are available on request from the corresponding author.
